# Significant Difference in Antimicrobial Resistance of Bacteria in Septic Revision between Total Knee Arthroplasty and Total Hip Arthroplasty

**DOI:** 10.3390/antibiotics11020249

**Published:** 2022-02-14

**Authors:** Stella Stevoska, Felix Himmelbauer, Julian Stiftinger, Christian Stadler, Lorenz Pisecky, Tobias Gotterbarm, Antonio Klasan

**Affiliations:** Department for Orthopaedics and Traumatology, Kepler University Hospital GmbH, Johannes Kepler University Linz, Krankenhausstrasse 9, 4020 Linz and Altenberger Strasse 69, 4040 Linz, Austria; himfel@gmail.com (F.H.); julian.stiftinger@kepleruniklinikum.at (J.S.); christian.stadler@kepleruniklinikum.at (C.S.); lorenz.pisecky@kepleruniklinikum.at (L.P.); tobias.gotterbarm@kepleruniklinikum.at (T.G.); antonio.klasan@kepleruniklinikum.at (A.K.)

**Keywords:** antimicrobial resistance, antibiotic, total knee arthroplasty, total hip arthroplasty, periprosthetic joint infections, revision

## Abstract

Antimicrobial resistance (AMR) aggravates the already difficult treatment of periprosthetic joint infections (PJI). Due to many factors influencing AMR, the correct choice of antimicrobial management remains arguable. The primary purpose of this retrospective study was to identify and compare bacteria and their antibiotic resistance profile between septic revision total knee arthroplasty (TKA) and septic revision total hip arthroplasty (THA). A review of all revision TKAs and revision THAs, undertaken between 2007 and 2020 in a tertiary referral hospital, was performed. Included were cases meeting the consensus criteria for PJI, in which an organism has been identified. There were no major differences in tissue sampling between revision TKAs and revision THAs over time. A total of 228 bacterial strains, isolated after revision TKA and THA, were analysed for their resistance to 20 different antibiotics. There was a statistically significant higher occurrence of Gram-negative bacteria identified after revision THAs compared to TKA (*p* = 0.002). The comparison of antibiotic resistance between revision TKAs and revision THAs was statistically significant in 9 of 20 analysed antibiotics. This has implications for the choice of empirical antibiotic in revision surgery as well as prophylactic antibiotic in primary surgery, depending on the joint that is to be replaced.

## 1. Introduction

The number of patients undergoing total knee arthroplasty (TKA) and total hip arthroplasty (THA) around the world is increasing due to higher life expectancy of the population, and further increase is expected [1,2]. Although the overall incidence of periprosthetic joint infection (PJI) is relatively low, 1% to 2% [3,4,5,6], it remains one of the biggest challenges and represents an enormous economic burden [7,8]. It occurs in approximately 0.97% of primary THA and 1.03% of primary TKA [6]. Eradication of an implant-associated infection is best achieved by a combination of both an appropriate surgical procedure and a prolonged antibiotic treatment, adjusted depending on antimicrobial susceptibility of the pathogen [9]. An emergence of antimicrobial resistance (AMR), especially to antibiotic classes commonly used in PJI, aggravates the already difficult treatment of PJI [3,10].

Antimicrobial resistance (AMR) has become one of the greatest global health issues of the 21st century [11]. It occurs naturally; however, excessive antibiotic use and misuse of antibiotics accelerates the process [12,13,14]. The prevalence of drug-resistant pathogens varies across countries [12,13,14] and changes over time [10,15,16]. Initiatives, such as ResistanceMap (resistancemap.org) and the World Health Organization’s (WHO) Global Antimicrobial Resistance Surveillance System (GLASS), document the geographic trends in antimicrobial resistance [12]. However, not all pathogens commonly isolated in PJI are included yet [12,13,14]. Studies focused on time-varying resistance mostly described an increase in AMR in PJI [15,16].

The most common bacteria causing periprosthetic joint infections are coagulase-negative staphylococci (CNS) in Europe [3,17,18,19,20] and Asia [21] and *Staphylococcus aureus* in the USA [20]. Additionally, there are microbiological differences reported depending on the joint infected [3,22,23,24]. Polymicrobial infections, anaerobes, and enteric Gram-negative bacteria are more commonly involved in the revision THA than in the revision of TKA [3,22,23,24,25]. In this context, only one study has descriptively compared antibiotic resistance incidence between revision TKA and revision THA [3]. The current literature comparing AMR regarding the joint location, therefore, remains scarce.

The aim of this retrospective clinical study was to identify and compare causative bacteria and to compare the incidence of antibiotic resistance between septic revision TKA and septic revision THA. Additionally, using a cluster analysis, two chronological time periods were created and the antibiotic resistance of the identified bacteria between these two clusters was evaluated.

## 2. Results

A total of 228 bacterial strains were analysed for their resistance—99 strains were isolated after revision TKA and 129 strains after revision THA. Coagulase-negative staphylococci (CNS) were the most common Gram-positive bacteria as well as the most common causative bacteria in both groups—in 56.6% (56 of 99 cultures) after revision TKAs and in 43.4% (56 of 129 cultures) after revision THAs. There was a statistically significant higher occurrence of Gram-negative bacteria identified after revision THAs compared to TKA (*p* = 0.002). This difference was mostly influenced by the occurrence of *Escherichia coli* after revision THAs, in 4.7% (6 of 129 cultures). Additionally, significantly higher occurrence of Gram-positive *Enteroccoccus* spp. after revision THA was detected (*p* = 0.026). On the contrary, an occurrence of *Streptococcus* spp. was significantly higher in revision TKA (*p* = 0.002) (Table 1).

The resistance occurrence after revision TKAs and THAs was analysed for a total of 20 different antibiotics: ampicillin, cefazolin, cefuroxime, ciprofloxacin, clindamycin, co-trimoxazole, erythromycin, Fosfomycin, fucidin acid, imipenem, levofloxacin, linezolid, moxifloxacin, oxacillin, penicillin, rifampicin, teicoplanin, tetracycline, tigecycline, vancomycin (Table 2).

In both groups, the strains were mostly resistant to penicillins, between 33.8% and 60.7% after revision TKAs and between 51.3% and 68.1% after revision THAs. In the THA group, high resistance was also detected against fluoroquinolones, between 42.6% and 61.9%, and against macrolide and erythromycin in 53.2%. Rifampicin resistance was observed in 22.2% in the THA group and only in 2.0% in the TKA group. There was almost no resistance to glycopeptides, teicoplanin, or vancomycin detected (Table 2).

The most effective antibiotics in the TKA group were glycopeptides-teicoplanin and vancomycin, imipenem, and linezolid, all with fully preserved sensitivity. Other antibiotics with almost entirely preserved sensitivity in the TKA group were tetracyclines and rifampicin (Table 2). Glycopeptides and linezolid were also very effective antibiotics in the THA group, with 98.8% and 98.9% sensitivity, respectively. However, the most sensitive antibiotic in the THA group was tigecycline with completely maintained sensitivity (Table 2).

The comparison of antibiotic resistance between revision TKAs and revision THAs was statistically significant in 9 of 20 analysed antibiotics: oxacillin (*p* = 0.03), ciprofloxacin (*p* < 0.001), levofloxacin (*p* < 0.001), moxifloxacin (*p* = 0.005), clindamycin (*p* < 0.001), co-trimoxazole (*p* < 0.001), imipenem (*p* = 0.01), rifampicin (*p* = 0.005), and tetracycline (*p* = 0.009). The difference in the resistance of strains was not statistically significant for ampicillin, penicillin, cefazolin, cefuroxime, erythromycin, Fosfomycin, fucidin acid, linezolid, tigecycline, teicoplanin, or vancomycin (Table 2).

Gram-positive bacteria isolated after the revision of THA were statistically significantly more resistant to ciprofloxacin (*p* < 0.001), levofloxacin (*p* < 0.001), moxifloxacin (*p* = 0.003), oxacillin (*p* = 0.03), and rifampicin (*p* = 0.002) (Table 3). If Gram-negative bacteria were isolated, a susceptibility to fluoroquinolones (ciprofloxacin, moxifloxacin), carbapenem (imipenem), co-trimoxazole, Fosfomycin, and tigecycline was primarily tested. No statistically significant difference in antibiotic resistance of Gram-negative bacteria was observed (Table 4).

For a total of 17 different antibiotics, the resistance occurrence between two chronological groups (90 and 138 strains) was compared (Table 5). There was a significant increase in bacteria resistance to rifampicin (2.8% to 18.6%, *p* = 0.02) and ciprofloxacin (29.2% to 45.2%, *p* = 0.03) (Table 5).

## 3. Discussion

The most common bacteria causing PJI in this study, carried out in a European tertiary referral hospital, were CNS, without any substantial difference between revision TKA and revision THA, 56.6% and 43.4%, respectively. However, the results of the present study show a statistically significantly higher occurrence of Gram-negative bacteria (*p* = 0.002) and *Enterococcus species* (*p* = 0.026) after revision THAs compared to revision TKAs (Table 1). In the present study, a statistically significant difference was detected in 9 of 20 analysed antibiotics. Pathogens isolated after revision THA were much more resistant compared to pathogens isolated after TKA. Resistance in revision THAs was significantly higher to oxacillin (*p* = 0.03), ciprofloxacin (*p* < 0.001), levofloxacin (*p* < 0.001), moxifloxacin (*p* = 0.005), clindamycin (*p* < 0.001), co-trimoxazole (*p* < 0.001), imipenem (*p* = 0.01), rifampicin (*p* = 0.005), and tetracycline (*p* = 0.009). There was no significantly higher resistance of pathogens isolated after revision TKAs detected (Table 2). No statistically significant difference in antibiotic resistance of Gram-negative bacteria between revision TKA and revision THA was observed (Table 4). Additionally, there was a statistically significant increase in antibiotic resistance of pathogens to rifampicin and ciprofloxacin observed (Table 5).

The proportion of detected CNS bacteria detected in the present study is comparable to the results reported in previous European studies, where CNS bacteria represented between 43.4% and 46.6% of isolated pathogens [3,10,15]. Gram-negative bacteria and *Enterococcus species*, isolated after THA, represent, with 18.6% and 10.9%, respectively, a substantial portion of causative pathogens in this study. Higher prevalence of enteric Gram-negative bacteria in revision THAs PJI may be due to its proximity to the gastrointestinal tract with the colonisation of enteric microorganisms around the hip and inguinal area [22,23].

Few other studies have focused on the comparison of microbiological profiles between revision TKA and revision THA. Tsai et al. [22] reported a statistically significant higher occurrence of anaerobes and polymicrobial pathogens in revision THA. In the present study, the occurrence of anaerobes after revision THA was not statistically significantly higher, however, it showed a greater tendency compared to revision TKA (Table 1). Preobrazhensky et al.’s [26] study also revealed changes in the distribution of microorganisms between revision TKA and revision THA. The proportions of *Staphylococcus aureus* and CNS were significantly higher in revision TKA and *Streptococcus* spp. in revision THA. On the contrary, Aggarwal et al. [20] and Drago et al. [3] found no significant differences in the microbiological profile between revision TKA and revision THA within each of their institutions. Nevertheless, Drago et al. [3], observed some differences in antibiotic resistance between revision TKA and revision THA, but did not statistically compare them.

On the other hand, studies regarding AMR differences between pathogens isolated after revision THA and revision TKA are scarce [3]. The present study showed an alarmingly high resistance of pathogens, isolated after revision THA, especially to fluoroquinolones (42.6% to 61.9%), a cornerstone therapy for Gram-negative PJI. The detected resistance to ciprofloxacin (50.5%) was much higher than the resistance recorded from Drago et al. (37.5%). Furthermore, the resistance to ciprofloxacin has statistically significantly increased in recent years (*p* = 0.03) (Table 5).

Currently, multi drug resistant (MDR) Gram-negative bacteria are on the rise around the globe [27], even in the orthopaedic field [28]. PJI due to Gram-negative pathogens is more difficult to treat and is associated with limited success [25,29]. Therefore, a comparison of the resistance of Gram-negative bacteria isolated after revision THA and revision TKA was additionally performed (Table 4). Resistance to fluoroquinolones, ciprofloxacin, and moxifloxacin, was higher after revision THA, 29.2% and 33.3%, respectively, compared to revision TKA, where no resistance was detected. The higher resistance of Gram-negative bacteria was, however, not statistically significant. Nevertheless, resistance to fluoroquinolones (e.g., ciprofloxacin) raises some concerns. There are still some alternative therapeutic strategies available. As such, there has been a resurgence of old antibiotics (e.g., Fosfomycin) and a development of a variety of new drugs and new drug combinations. Another area of promising research, also in the orthopaedic field, is a resurgence of an older antimicrobial treatment strategy: bacteriophage therapy [27]. In the present study, a low bacterial resistance of Gram-negative bacteria to carbapenems (imipenem), 9.5%, is still maintained. However, the resistance of Gram-negative bacteria to Fosfomycin is high, 66.7% after revision TKA and 38.5% after revision THA (Table 4). Therefore, an alternative therapy with Fosfomycin in the analysed referral hospital, does not seem like a viable option.

As in previous studies [3,10], a relevantly high resistance to the penicillin group of antibiotics was detected (Table 2). Pathogens isolated after THA revision were significantly more resistant to oxacillin/methicillin (*p* = 0.03), in 51.3%, compared to after TKA revision, in 33.8%. The methicillin resistant bacteria do not respond to cefuroxime, cefazolin or any of the other numerous b-lactam drugs [30,31]. Although a cefuroxime sensitivity seems to be maintained, it was not as commonly tested as oxacillin (Table 2). Therefore, in patients, who are carriers of methicillin resistant bacteria, a prophylactic antibiotic treatment with glycopeptides, such as vancomycin and teicoplanin, can be considered. Both showed maintained susceptibility in revision TKA and revision THA in the present study (Table 2). However, an improper or unnecessary widespread use carries the risk of developing glycopeptide resistance [3,31,32] and is, therefore, not widely supported. Nevertheless, vancomycin use as an empirical antibiotic or as a part of dual empirical antibiotic therapy, at least in revision THA, where methicillin-resistant pathogens seem to be more common, remains reasonable.

Rifampicin, as a biofilm active antibiotic, plays a major role in the treatment of staphylococcal periprosthetic joint infections [4,9,10,33]. A significantly higher resistance to rifampicin after revision THA, 22.2%, compared to resistance of only 2.0% after revision TKA in this referral hospital, is worrying. On the other hand, Drago et al. reported a higher rifampicin resistance after revision TKA (between 12.5% and 30.5%) compared to revision THA (between 10.4% and 21.2%) [3]. The risk of developing rifampicin resistance is known to be high due to its structure and incorrect clinical use [33,34]. Such infections are difficult to eradicate and with statistically significant increasing rifampicin resistance (*p* = 0.02), as reported in this study, fewer patients with PJI would be able to benefit from orthopaedic implant retention (Table 5).

This study has several limitations. Owing to its retrospective design, there is a collection and selection bias. Due to a wide catchment area, the exact history of each patient could not be accounted for. Any previous surgical or antibiotic treatment that was used outside of in this study investigated institutions is unknown and, therefore, the potential influence of this on the resistance was not reviewed. Additionally, the study was conducted at a single referral hospital, which may cause selection bias. Moreover, there might have been changes in microbiology protocols in the included years unknown to authors of this study. Secondly, polymicrobial pathogens were not excluded and were not separately analysed. Additionally, not every isolated pathogen was tested on every antibiotic included in this study. For example, if Gram-negative bacteria were isolated, a susceptibility to fluoroquinolones, carbapenem, co-trimoxazole, Fosfomycin, and tigecycline was primarily tested. Finally, the study was conducted on the groups of causative pathogens and, therefore, lacking on data about antibiotic resistance of single species.

## 4. Methods

This retrospective, single-centre cohort study included all revision TKAs and revision THAs undertaken between 2007 and 2020 in a tertiary referral hospital. The study was approved by the University’s Ethics Board (1253/2020). Included were all cases meeting the consensus criteria for PJI, in which an organism has been identified [35]. Retrospective information was collected on the choice of empiric antibiotic treatment and microbiology. The microbiological database was searched for the pathogens detected intraoperatively and for antimicrobial susceptibility testing.

### 4.1. Surgical Treatment

All patients presenting with possible PJI due to an elevated CRP and/or signs of local inflammation underwent a sterile aspiration of synovial fluid and microbiological analysis. No later than six weeks after the aspiration, the revision procedure was performed. All the revisions were either two-stage or multiple-stage procedures. In the first stage, all components, including cement, if present, were removed and multiple deep intra-operative tissue samples from different areas were obtained. After an aggressive debridement and irrigation, a static or mobile antibiotic-loaded cement spacer was implanted. Systemic antibiotics were administered for six weeks. In patients with low CRP < 20 mg/L and no local signs of infection, the second stage, where new components and renewed postoperative antibiotic therapy were introduced, followed. If there was any suspicion of persistent infection, a repeat debridement with spacer exchange was undertaken. The technique for performing revision TKA and THA for PJI remained the same throughout the study period.

### 4.2. Microbiology

Routinely, at each stage, at least three tissue samples and one sample of synovial fluid were obtained for culture. Synovial fluid was aseptically inoculated into aerobic and anaerobic blood culture bottles (BACTEC, Beckton Dickinson, Franklin Lakes, NJ, USA) and incubated for 14 days. Tissue samples were obtained from the subfascial tissue and the proximal and distal interfaces of the prosthesis. Each culture was transported, processed, and analysed according to international standards and the definitions of the European Committee on Antimicrobial Susceptibility Testing (EUCAST) [36]. Tissues were inoculated onto Columbia agar, chocolate agar, McConkey agar and Schädler agar and into Brain-Heart-Infusion (BHI) broth. The broth was subcultured the next day. All culture media were incubated for 14 days and inspected every day for bacterial growth. Antibiotic susceptibility testing was performed using the disk-diffusion technique and presented according to the EUCAST criteria that were valid at the time of testing.

Additionally, sonication of the explanted prosthetic components was performed. The explanted components were placed in sterile polypropylene containers and in the laboratory immersed in Ringer’s solution. After 5 min of vigorous manual shaking, explanted prosthetic components were sonicated for 5 min followed by an additional 5 min of vigorous shaking. 500 µL were directly applied onto Columbia agar and Schädler agar. 50 mL of sonication fluid was centrifuged at 3000 U for 5 min. The supernatant was aspirated, and the sediment was inoculated into BHI broth. All culture media were incubated for 14 days and inspected every day for bacterial growth.

Oxacillin resistance was used to classify organisms as resistant to methicillin. There were no major differences in tissue sampling between revision TKAs and revision THAs over time.

### 4.3. Antibiotic Treatment

The chosen prophylactic antibiotic used before the primary non-revision surgery was a single shot of 1500 mg intravenous cefuroxime. In the case of the history of penicillin and/or cephalosporin hypersensitivity, antibiotic prophylaxis has been adapted accordingly and was usually replaced by a single preoperative shot of clindamycin.

In revision surgery, in case of suspected PJI, an intravenous antibiotic therapy of wide antimicrobial-spectrum was started intraoperatively directly after tissue sampling. The usual empiric preoperative antibiotic therapy used was ampicillin/sulbactam. In septic patients, known MRSA-carriers, or patients with suspected low-grade infection, vancomycin, as a monotherapy or as an addition to ampicillin/sulbactam was usually initiated. Once pathogens were known, and the antimicrobial susceptibility was available, antibiotic therapy was adapted to the resistance situation in collaboration with microbiologists. If possible, rifampicin, effective against biofilm, was added to the treatment in cases of infection with Gram-positive bacteria as soon as the skin wound showed no drainage. In case of resistance to rifampicin, or in the case of isolation of Gram-negative bacteria, a further therapy was determined individually with the microbiologists for each patient.

### 4.4. Outcome Measures

The main outcome measure of this retrospective study was to identify and compare bacteria and their antibiotic resistance profile between septic revision TKA and septic revision THA. Additionally, using a cluster analysis, two chronological time periods were created and the antibiotic resistance of the identified bacteria between these two clusters was evaluated.

### 4.5. Statistical Analysis

Data were collected using Microsoft Excel (Redmond, WA, USA) and analysed with SPSS v.24 (IBM, Armonk, New York, NY, USA). The study cohort was described using descriptive statistics. Overall antibiotic resistance was calculated by determining the number of antibiotics to which each culture was sensitive. A formal power analysis using an alfa level of 0.05 and beta level of 0.8 estimated the minimum sample size of at least 15 cultures per group. Antibiotics with insufficient analysis power were excluded. Chronological groups were created using a two-step cluster analysis. First, a hierarchical cluster analysis of the number of cases per year was performed to determine the number of clusters, after which k-means cluster analysis was performed to identify the cases in each cluster. The differences between the groups were analysed using the chi-squared test. A *p*-value of < 0.05 was considered statistically significant.

## 5. Conclusions

The occurrence and the resistance of bacteria to antibiotics differs significantly between revision TKAs and revision THAs. Based on these findings, a referral hospital should continuously monitor their microbiological cultures, differentiating between the hips and knees, as this has significant implications on the choice of empirical antibiotic in revision surgery as well as prophylactic antibiotics in primary surgery, depending on the joint that is to be replaced.

## Figures and Tables

**Table 1:** List of bacteria identified in the cultures.

Bacteria	Occurrence in TKA: *n* (%)	Occurrence in THA: *n* (%)	*p*-Value (Significant < 0.05)
**Gram-positive**	**94 (94.9)**	**105 (81.4)**	***p* = 0.002**
**CPS**	**16 (16.2)**	**23 (17.8)**	***p* = 0.740**
*Staphylococcus aureus*	15 (15.2)	22 (17.1)	
MRSA	0	1 (0.8)	
*Staphylococcus pseudintermedius*	1 (1.0)	0	
**CNS**	**56 (56.6)**	**56 (43.4)**	***p* = 0.049**
*Staphylococcus capitis*	1 (1.0)	3 (2.3)	
*Staphylococcus caprae*	1 (1.0)	0	
*Staphylococcus cohnii*	1 (1.0)	0	
*Staphylococcus epidermidis*	42 (42.4)	42 (32.6)	
*Staphylococcus haemolyticus*	3 (3.0)	8 (6.2)	
*Staphylococcus hominis*	4 (4.0)	0	
*Staphylococcus lugdunensis*	3 (3.0)	2 (1.6)	
*Staphylococcus saccharolyticus*	0	1 (0.8)	
*Staphylococcus saprophyticus*	1 (1.0)	0	
***Streptococcus* spp.**	**13 (13.1)**	**3 (2.3)**	***p* = 0.002**
*Streptococcus agalactiae*	3 (3.0)	2 (1.6)	
*Streptococcus gordonii*	1 (1.0)	0	
*Streptococcus oralis*	1 (1.0)	0	
β-haemolytic streptococci group G	8 (8.0)	1 (0.8)	
**Anaerobes**	**3 (3.0)**	**9 (7.1)**	***p* = 0.190**
*Propionibacterium*	3 (3.0)	6 (4.7)	
*Cutibacterium acnes*	0	1 (0.8)	
*Finegoldia magna*	0	1 (0.8)	
*Peptostreptococcus magnus*	0	1 (0.8)	
***Enterococcus* spp.**	**3 (3.0)**	**14 (10.9)**	***p* = 0.026**
*Enterococcus faecalis*	2 (2.0)	13 (10.1)	
*Enterococcus faecium*	1 (1.0)	1 (0.8)	
*Corynebacterium*	3 (3.0)	0	
**Gram-negative**	**5 (5.1)**	**24 (18.6)**	***p* = 0.002**
*Acinetobacter baumannii*	0	1 (0.8)	
*Citrobacter koseri*	0	2 (1.6)	
*Enterobacter cloacae*	2 (2.0)	3 (2.3)	
*Escherichia coli*	1 (1.0)	6 (4.7)	
*Klebsiella pneumoniae*	0	4 (3.1)	
*Klebsiella variicola*	0	1 (0.8)	
*Morganella morganii*	0	2 (1.6)	
*Pseudomonas aeruginosa*	2 (2.0)	5 (3.9)	
**Total number**	**99**	**129**	

CPS, coagulase-positive staphylococci; MRSA, methicillin-resistant *Staphylococcus aureus*; CNS, coagulase-negative staphylococci; *n,* number; TKA, total knee arthroplasty; THA, total hip arthroplasty.

**Table 2:** Antibiotic resistance of 20 different types of antibiotics and its statistical significance, comparing revision TKA and revision THA.

Type of Antibiotic	TKA (*n* = 99)		THA (*n* = 129)		*p*-Value (Significant < 0.05)
	S	R	S	R	
**Penicillin grp.**					
Ampicillin	14/30 (46.7%)	16/30 (53.3%)	14/35 (40.0%)	21/35 (60.0%)	0.751
Oxacillin	49/74 (66.2%)	25/74 (33.8%)	39/80 (48.8%)	41/80 (51.3%)	0.03
Penicillin	35/89 (39.3%)	54/89 (60.7%)	29/91 (31.9%)	62/91 (68.1%)	0.3
**Cephalosporins**					
Cefazolin	14/21 (66.7%)	7/21 (33.3%)	11/17 (64.7%)	6/17 (35.3%)	0.270
Cefuroxime	20/27 (74.1%)	7/27 (25.9%)	12/20 (60.0%)	8/20 (40.0%)	0.055
**Fluoroquinolones**					
Ciprofloxacin	62/82 (75.6%)	20/82 (24.4%)	52/105 (49.5%)	53/105 (50.5%)	<0.001
Levofloxacin	24/31 (77.4%)	7/31 (22.6%)	24/63 (38.1%)	39/63 (61.9%)	<0.001
Moxifloxacin	66/85 (77.7%)	19/85 (22.3%)	58/101 (57.4%)	43/101 (42.6%)	0.005
**Lincosamide**					
Clindamycin	60/90 (66.7%)	30/90 (33.3%)	47/89 (52.8%)	42/89 (47.2%)	<0.001
**Sulfonamide**					
Co-trimoxazole	32/40 (80.0%)	8/40 (20.0%)	59/90 (65.6%)	31/90 (34.4%)	<0.001
**Macrolide**					
Erythromycin	36/62 (58.1%)	26/62 (41.9%)	36/77 (46.8%)	41/77 (53.2%)	0.373
**Phosphonic acid**					
Fosfomycin	56/76 (73.7%)	20/76 (26.3%)	56/92 (60.9%)	36/92 (39.1%)	0.138
**Fusidane class**					
Fusidic acid	57/74 (77.0%)	17/74 (23.0%)	60/80 (75.0%)	20/80 (25.0%)	0.121
**Carbapenem**					
Imipenem	15/15 (100.0%)	0/15 (0.0%)	38/41 (92.7%)	3/41 (7.3%)	0.010
**Oxazolidinone**					
Linezolid	63/63 (100.0%)	0/63 (0.0%)	88/89 (98.9%)	1/89 (1.1%)	0.490
**Antimycobacterial**					
Rifampicin	49/50 (98.0%)	1/50 (2.0%)	56/72 (77.8%)	16/72 (22.2%)	0.005
**Tetracycline grp.**					
Tetracycline	35/36 (97.2%)	1/36 (2.8%)	64/72 (88.9%)	8/72 (11.1%)	0.009
Tigecycline	61/62 (98.4%)	1/62 (1.6%)	102/102 (100.0%)	0/102 (0.0%)	0.015
**Glycopeptides**					
Teicoplanin	60/60 (100.0%)	0/60 (0.0%)	82/83 (98.8%)	1/83 (1.2%)	0.590
Vancomycin	72/72 (100.0%)	0/72 (0.0%)	85/86 (98.8%)	1/86 (1.2%)	0.403

TKA, total knee arthroplasty; THA, total hip arthroplasty; *n*, number; S, sensitive; R, resistant; grp., group.

**Table 3:** Antibiotic resistance of 20 different types of antibiotics to Gram-positive bacteria and its statistical significance, comparing revision TKA and revision THA.

Type of Antibiotic	TKA (*n* = 94)		THA (*n* = 105)		*p*-Value (Significant < 0.05)
	S	R	S	R	
Ampicillin	14/27 (51.9%)	13/27 (48.1%)	13/17 (76.5%)	4/17 (23.5%)	0.10
Oxacillin	49/74 (66.2%)	25/74 (33.8%)	39/80 (48.8%)	41/80 (51.3%)	0.03
Penicillin	35/89 (39.3%)	54/89 (60.7%)	29/91 (31.9%)	62/91 (68.1%)	0.3
Cefazolin	14/20 (70.0%)	6/20 (30%)	11/17 (64.7%)	6/17 (35.3%)	0.73
Cefuroxime	18/24 (75.0%)	6/24 (25.0%)	3/5 (60%)	2/5 (40%)	0.49
Ciprofloxacin	57/77 (74.0%)	20/77 (15.4%)	35/81 (43.2%)	46/81 (56.8%)	<0.001
Levofloxacin	23/29 (79.3)	6/29 (20.7%)	24/63 (38.1%)	39/63 (61.9%)	<0.001
Moxifloxacin	65/84 (77.4%)	19/84 (22.6%)	46/83 (55.4%)	37/83 (44.6%)	0.003
Clindamycin	60/90 (66.7%)	30/90 (33.3%)	47/89 (52.8%)	42/89 (47.2%)	0.06
Co-trimoxazole	30/37 (81.1%)	7/37 (18.9%)	46/71 (64.8%)	25/71 (35.2%)	0.08
Erythromycin	36/61 (59.0%)	25/61 (41.0%)	36/77 (46.8%)	41/77 (53.2%)	0.15
Fosfomycin	55/73 (75.3%)	18/73 (24.7%)	48/79 (60.8%)	31/79 (39.2%)	0.06
Fucidin acid	57/74 (77.0%)	17/74 (23.0%)	60/80 (75.0%)	20/80 (25.0%)	0.77
Imipenem	10/10 (100.0%)	0/10 (0.0%)	19/20 (95.0%)	1/20 (5.0%)	0.47
Linezolid	62/62 (100.0%)	0/62 (0.0%)	88/89 (98.9%)	1/89 (1.1%)	0.4
Rifampicin	48/49 (98.0%)	1/49 (2.0%)	56/72 (77.8%)	16/72 (22.2%)	0.002
Tetracycline	34/35 (97.1%)	1/35 (2.9%)	64/72 (88.9%)	8/72 (11.1%)	0.15
Tigecycline	60/60 (100.0%)	0/60 (0.0%)	89/89 (100.0%)	0/89 (0.0%)	NA
Teicoplanin	59/59 (100.0%)	0/59 (0.0%)	82/83 (98.8%)	1/83 (1.2%)	NA
Vancomycin	71/71 (100.0%)	0/71 (0.0%)	85/86 (98.8%)	1/86 (1.2%)	0.36

TKA, total knee arthroplasty; THA, total hip arthroplasty; *n*, number; S, sensitive; R, resistant; NA, not available.

**Table 4:** Antibiotic resistance of 20 different types of antibiotics to Gram-negative bacteria and its statistical significance, comparing revision TKA and revision THA.

Type of Antibiotic	TKA (*n* = 5)		THA (*n* = 24)		*p*-Value (Significant < 0.05)
	S	R	S	R	
Ampicillin	0/3 (0.0%)	3/3 (100.0%)	1/18 (5.6%)	17/18 (94.4%)	0.68
Oxacillin	NA	NA	NA	NA	NA
Penicillin	NA	NA	NA	NA	NA
Cefazolin	0/1 (0.0%)	1/1 (100.0%)	NA	NA	NA
Cefuroxime	2/3 (66.7%)	1/3 (33.3%)	9/15 (60%)	6/15 (40%)	0.83
Ciprofloxacin	5/5 (100.0%)	0/5 (0.0%)	17/24 (70.8%)	7/24 (29.2%)	0.17
Levofloxacin	1/2 (50.0%)	1/2 (50.0%)	NA	NA	NA
Moxifloxacin	1/1 (100.0%)	0/1 (0.0%)	12/18 (66.7%)	6/18 (33.3%)	0.49
Clindamycin	NA	NA	NA	NA	NA
Co-trimoxazole	2/3 (66.7%)	1/3 (33.3%)	13/19 (68.4%)	6/19 (31.6%)	0.95
Erythromycin	0/1 (0.0%)	1/1 (100.0%)	NA	NA	NA
Fosfomycin	1/3 (33.3%)	2/3 (66.7%)	8/13 (61.5%)	5/13 (38.5%)	0.37
Fucidin acid	NA	NA	NA	NA	NA
Imipenem	5/5 (100.0%)	0/5 (0.0%)	19/21 (90.5%)	2/21 (9.5%)	NA
Linezolid	1/1 (100.0%)	0/1 (0.0%)	NA	NA	NA
Rifampicin	1/1 (100.0%)	0/1 (0.0%)	NA	NA	NA
Tetracycline	1/1 (100.0%)	0/1 (0.0%)	NA	NA	NA
Tigecycline	1/2 (50.0%)	1/2 (50.0%)	13/13 (100.0%)	0/13 (0.0%)	0.01
Teicoplanin	1/1 (100.0%)	0/1 (0.0%)	NA	NA	NA
Vancomycin	1/1 (100.0%)	0/1 (0.0%)	NA	NA	NA

TKA, total knee arthroplasty; THA, total hip arthroplasty; *n*, number; S, sensitive; R, resistant; NA, not available.

**Table 5:** Antibiotic resistance of 17 different types of antibiotics and the statistical significance, comparing two chronological groups.

Type of Antibiotic	2007–2016 (*n* = 90)		2017–2020 (*n* = 138)		*p*-Value (Significant < 0.05)
	S	R	S	R	
**Penicillin grp.**					
Ampicillin	14/33 (42.4%)	19/33 (57.6%)	14/32 (43.8%)	18/32 (56.2%)	0.91
Oxacillin	40/67 (59.7%)	27/67 (40.3%)	48/87 (55.2%)	39/87 (44.8%)	0.57
Penicillin	33/81 (40.7%)	48/81 (59.3%)	31/99 (31.3%)	68/99 (68.7%)	0.19
**Cephalosporins**					
Cefuroxime	21/30 (70.0%)	9/30 (30.0%)	11/17 (64.7%)	6/17 (35.3%)	0.71
**Fluoroquinolones**					
Ciprofloxacin	51/72 (70.8%)	21/72 (29.2%)	63/115 (54.8%)	52/115 (45.2%)	0.03
Moxifloxacin	57/77 (74.0%)	20/77 (26.0%)	67/109 (61.5%)	42/109 (38.5%)	0.07
**Lincosamide**					
Clindamycin	52/80 (65.0%)	28/80 (35.0%)	55/99 (55.6%)	44/99 (44.4%)	0.20
**Sulfonamide**					
Co-trimoxazole	20/29 (69.0%)	9/29 (31.0%)	71/101 (70.3%)	30/101 (29.7%)	0.89
**Macrolide**					
Erythromycin	26/51 (51.0%)	25/51 (49.0%)	46/88 (52.3%)	42/88 (47.7%)	0.88
**Phosphonic acid**					
Fosfomycin	45/70 (64.3%)	25/70 (35.7%)	67/98 (68.4%)	31/98 (31.4%)	0.58
**Fusidane class**					
Fusidic acid	53/67 (79.1%)	14/67 (20.9%)	64/87 (73.6%)	23/87 (26.4%)	0.43
**Oxazolidinone**					
Linezolid	49/49 (100.0%)	0	102/103 (99.0%)	1 (1.0%)	0.49
**Antimycobacterial**					
Rifampicin	35/36 (97.2%)	1/36 (2.8%)	70/86 (81.4%)	16/86 (18.6%)	0.02
**Tetracycline grp.**					
Tetracycline	23/24 (95.8%)	1/24 (4.2%)	76/84 (90.5%)	8/84 (9.5%)	0.40
Tigecycline	51/52 (98.1%)	1 (1.9%)	112/112 (100.0%)	0	0.14
**Glycopeptides**					
Teicoplanin	48/48 (100.0%)	0	94/95 (99.0%)	1/95 (1.0%)	0.48
Vancomycin	57/57 (100.0%)	0	100/101 (99.0%)	1/101 (1.0%)	0.45

S, sensitive; R, resistant; *n*, number; grp., group.

## Data Availability

The data presented in this study are available upon request from the corresponding author.
